# Different immune responses to three different vaccines following H6N1 low pathogenic avian influenza virus challenge in Taiwanese local chicken breeds

**DOI:** 10.1186/1753-6561-5-S4-S33

**Published:** 2011-06-03

**Authors:** Chi-Sheng Chang, Michèle Tixier-Boichard, Olympe Chazara, Yen-Pai Lee, Chih-Feng Chen, Poa-Chun Chang, Jan-Wei Chen, Bertrand Bed’hom

**Affiliations:** 1 Department of Animal Science, National Chung-Hsing University, Taiwan, R.O.C; 2 Graduate Institute of Microbiology and Public Health, National Chung-Hsing University, Taiwan, R.O.C; 3 Institute of Biomedical Science, National Chung-Hsing University, Taiwan, R.O.C; 4 UMR1313 Génétique Animale et Biologie Intégrative, INRA/AgroParisTech, Jouy-en-Josas, France

## Abstract

**Background:**

H6N1 low pathogenic avian influenza virus (LPAIV) are frequently isolated in Taiwan and lead to significant economic losses, either directly or indirectly through association with other infectious diseases. This study investigates immune responses to three different vaccines following a H6N1 challenge in different local breeds.

**Methods:**

Experimental animals were sampled from six local chicken breeds maintained at the National Chung-Hsing University, namely Hsin-Yi, Ju-Chi, Hua-Tung (Taiwan), Quemoy (Quemoy Island), Shek-Ki (China), Nagoya (Japan) and a specific pathogen free (SPF) White Leghorn line. A total number of 338 chickens have been distributed between a control and a challenge group, H6N1 challenge was performed at 7 weeks of age; vaccination against Newcastle Disease (ND), Infectious Bursal Disease (IBD) and Infectious Bronchitis (IB) was performed at 11 weeks. The anti-H6N1 LPAIV antibody titers were measured by ELISA at days 0, 7, 14 and 21 after challenge, and the anti-ND, anti-IBD and anti-IB antibody titers were measured by inhibition of hemagglutination test and ELISA at days 0, 14, 28 after vaccination.

**Results:**

There was no effect of the H6N1 LPAIV challenge at 7 weeks of age on the subsequent responses to ND and IBD vaccine at 11 weeks of age, but, surprisingly, the H6N1 LPAIV challenge significantly affected antibody levels to IB vaccine in some breeds, since IB0 and IB14 antibody titers were lower in the challenge groups. However, there was no significant difference in IB28 antibody titers among the experimental groups.

**Conclusions:**

Local breeds have different immune response to H6N1 LPAIV challenge and subsequent vaccines. Differences dealt mainly with kinetics of response and with peak values. Quemoy exhibited higher antibody levels to H6N1, ND and IBD. The negative effect of the H6N1 LPAIV challenge on IB vaccine response may be related to the fact that both viruses target the lung tissues, and the type of local immune response induced by LPAIV challenge may not be favourable for birds to make optimum IB-specific antibody response.

## Background

Since 1982, National Chung-Hsing University is maintaining six local chicken breeds: Hsin-Yi, Ju-Chi and Hua-Tung were collected from small villages in Taiwan, Quemoy was collected from Quemoy Island near China, Shek-Ki was from China, and Nagoya was from Japan [1]. In previous studies, Quemoy had significantly higher antibody titers against Newcastle Disease (ND) after vaccination than other local breeds, Shek-Ki, Hua-Tung and Ju-Chi had lower anti-ND antibody titers [2]. Thus, immune response was shown to differ within this set of local chicken breeds.

H6N1 Low pathogenic Avian Influenza Virus (LPAIV) is frequently isolated in Taiwan and lead to significant economic losses, either directly or indirectly through association with other infectious diseases [3]. This study investigated immune responses to ND, Infectious Bronchitis (IB) and Infectious Bursal Disease (IBD) vaccines following a H6N1 LPAIV challenge on six local chicken breeds and SPF chicken.

## Methods

### Experimental chickens

In this study we used the six local breeds and added an SPF White Leghorn as a control genotype.

A total of 314 chicks were hatched from 23 sires and 91 dams with full pedigree in six local breeds. Twenty-five SPF chicks were purchased from Animal Health Research Institute (Council of Agriculture, Executive Yuan R.O.C.). Day-old chicks were wing-banded and raised in floor pens until 5 weeks of age, and they were transferred to experimental cages after 6 weeks of age. Sire families were distributed between the control and the challenge group. Individual body weights were recorded weekly from hatch to 16 weeks of age.

### Vaccination program and challenge

Day-old chicks were all vaccinated against Marek’s disease and ND. At two weeks of age, chicks were vaccinated against ND, IB, IBD, Fowl Pox and Avian Reovirus infection. At four weeks of age, chickens were vaccinated against ND, IB, IBD and Infectious Laryngotracheitis. The H6N1 LPAIV (A/chicken/Taiwan/0825/2006) challenge was performed at 7 weeks of age, birds from the challenge group received a drop with 10^7^ EID_50_ of viruses into eye and nose. Blood samples were collected at days 0, 7, 14 and 21 post-challenge. Chickens health condition and mortality were recorded and monitored. At 11 weeks of age, all chickens, from both challenge and control groups, were vaccinated again against ND, IB and IBD, blood samples were collected at days 0, 14 and 28 post-inoculation. Sera from blood samples were collected and stored in -20 *°*C refrigerator.

### Immune response measurement

The antibody responses to H6N1 LPAIV, IBD and IB were measured by enzyme-linked immunosorbent assay (ELISA) with commercial test kit (IDEXX Laboratories, Inc., Westbrook, ME), the antibody titer calculation was performed according to IDEXX’s formula. The antibody responses to ND were measured by hemagglutination inhibition test (HIT), the antibody titer was expressed as the log2 of the reciprocal of the highest dilution. Each measure was defined by the virus name (AI, ND, IBD or IB) and the day of sampling after inoculation (either days 0, 7, 14, 21 or 28). In addition, response to IB vaccine was calculated by the difference in antibody titers between stages, i.e. day 0 to day 14, day 14 to day 28 and day 0 to day 28.

### Statistical analysis

Antibody titers and differences between successive titers were analysed with the following statistical model,

where *Y_ijkl_* is the antibody titer of the *l*th animal of the *i*th breed, the *k*th sex after the *j*th challenge treatment, *i*=1, 2,…,7, *j*=1,2, *k*=1,2, *l*=1,2,…,338, *μ* is the mean, *τ_i_* is the fixed effect of the *i*th breed, Σ*τ_i_*=0, *α_j_* is the fixed effect of the *j*th challenge treatment, Σ*α_j_*=0, (*τα*)*_ij_* is the fixed interaction effect between the *i*th breed and the *j*th challenge treatment , ΣΣ(*τα*)*_ij_*=0, and *e_ijkl_* is the residual random error, .

All statistical analyses were conducted by using SAS software (SAS Institute).

## Results

### H6N1 LPAIV challenge

**Figure 1:**
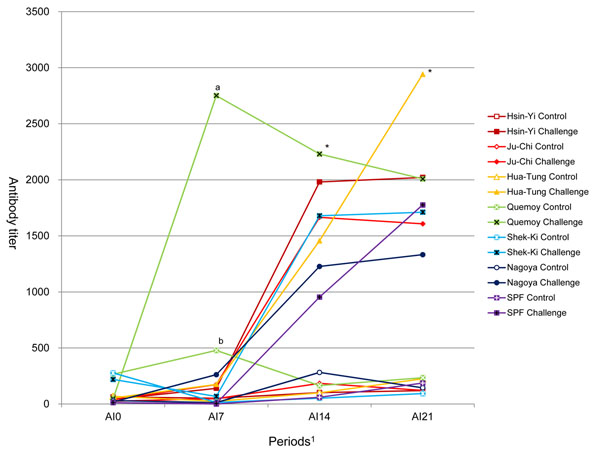
**Antibody titers against H6N1 LPAIV on 0, 7, 14 and 28 days post-challenge. **^a,b^ within days post-challenge with no common superscript differ significantly (*P* < 0.05) in Quemoy. ^*^ means all breeds have significantly different between control and challenge group.

There was no mortality for Quemoy, Nagoya and SPF chickens (Table 1). Ju-Chi, Hua-Tung and Shek-Ki exhibited a low mortality (one bird each), and Hsin-Yi had the highest mortality: 7 birds (22.6%, see Table 1). Most of mortality occurred between day 7 and 14 post-challenge. The analysis of variance showed a significant effect of breed on all antibody titers, no effect of sex, and a significant effect of the challenge with a significant breed with challenge interaction on antibody titers from day 7 (Fig 1). This interaction was due to differential response between breeds: Quemoy had the highest antibody titer on day 7 and 14 post-challenge, Hua-Tung showed the highest antibody titer on day 21 post-challenge, Nagoya and SPF showed lower antibody titer on day 14 and 21 post-challenge. Quemoy was the only breed to exhibit a significant antibody titer at day 7 post-challenge. The anti-AI antibody titers were significantly higher for all breeds at days 14 and 21 post-challenge.

**Table 1:** Sample size and mortality per breed in H6N1 challenge experiment

	Hsin-Yi	Ju-Chi	Hua-Tung	Quemoy	Shek-Ki	Nagoya	SPF
Control group	31	25	23	42	9	15	12
Challenge group	31	27	28	49	12	22	12
Total size	62	52	51	91	21	37	24
Mortality^*^	7	1	1	0	1	0	0
Mortality rate (%)	22.6	3.7	3.6	0	8.3	0	0

^*^ Mortality was calculated within challenge group.

### ND vaccine response

**Figure 2:**
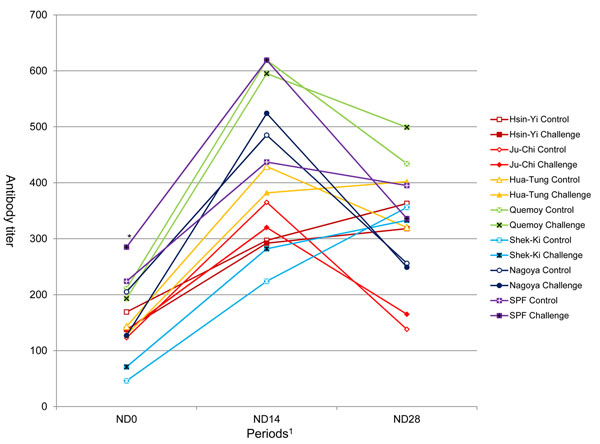
Antibody titers against ND on 0, 14 and 28 days post-inoculation. ^*^ Nagoya showed a difference of anti-ND antibody titer between the control and challenge groups at day 0, with a lower value in the challenge group.

There was no effect of the H6N1 LPAIV challenge on anti-ND antibody titers at day 14 and 28 post-inoculation (Fig 2). Nagoya was the only one to show a difference of anti-ND antibody titer between the control and challenge groups at day 0, with a lower value in the challenge group. The breed effect was significant at all stages and the sex effect was not (data not shown). The Quemoy and SPF had high antibody levels from ND0 to ND28. Ju-Chi showed the lowest response to ND vaccination at day 28 post-inoculation.

### IBD vaccine response

**Figure 3:**
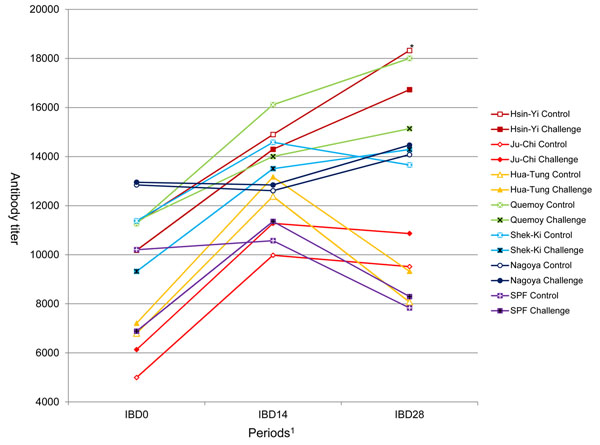
Antibody titers against IBD on 0, 14 and 28 days post-inoculation. ^*^ Quemoy showed significantly lower antibody titer in the challenge group.

There was a significant effect of breed and no effect of the H6N1 LPAIV challenge on anti-IBD antibody titers at all stages. Interaction between breed and treatment tended to be significant at day 28 (*P* < 0.05) where the Quemoy was the only one to show significantly lower antibody titers in the challenge group (Fig 3). There was a significant sex effect on responses at days 14 and 28 post-inoculation, and the antibody titers were higher in females than in males (data not shown). Nagoya and SPF showed no response to vaccination, but antibody titers of Nagoya were rather high at day 0. Quemoy and Hsin-Yi showed the highest antibody levels, particularly at day 28 post-inoculation. Ju-Chi, Hua-Tung and SPF showed the lowest antibody titers at all stages.

### IB vaccine response

**Figure 4:**
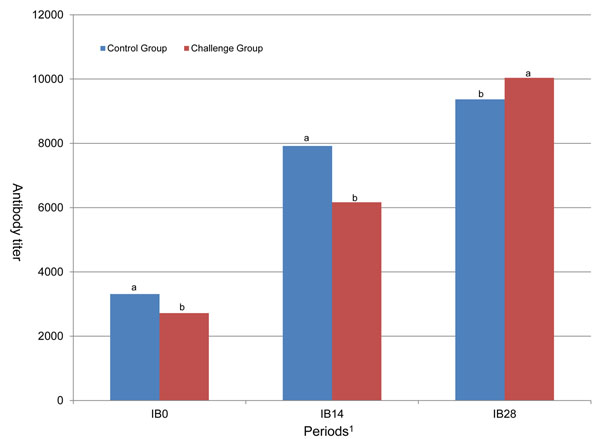
**Antibody titers against IB on 0, 14 and 28 days post-inoculation. **^a,b^ within days post-inoculation with no common superscript differ significantly (*P* < 0.05).

**Figure 5:**
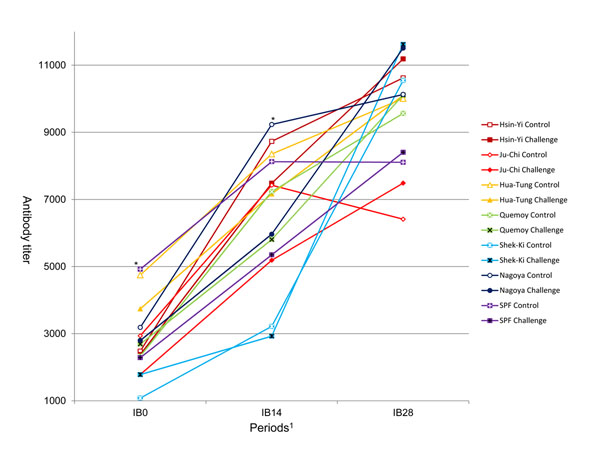
Antibody titers against IB on 0, 14 and 28 days post-inoculation. ^*^ Antibody titers at day 0 were lower in the challenge group than in the control group for Ju-Chi and SPF. The effects of breed and H6N1 challenge, without any interaction, were still observed at day 14 post-inoculation. Higher antibody levels were found in the control group for all breeds. Nagoya was the only one to exhibit a stronger response to IB vaccine in the H6N1 control group at day 14.

In contrast with the results observed for ND and IBD, the kinetics of antibody titers of IB was modified by the H6N1 LPAIV challenge (Fig 4). Interactions between breed and treatment, as well as between breed and sex, were significant for IB0. Antibody titers at day 0 were lower in the challenge group than in the control group for Ju-Chi and SPF, but did not differ between groups for the other breeds (Fig 5). The effects of breed and H6N1 challenge, without any interaction, were still observed at day 14 post-inoculation. Higher antibody levels were found in the control group for all breeds. Nagoya was the only one to exhibit a stronger response to IB vaccine in the H6N1 control group at day 14, as measured by the difference between titers at day 14 and day 0 (Table 2). The interaction between breed and sex was still significant at day 14 but was not observed at day 28. Breed and treatment effects were significant at day 28; antibody titers became higher in the challenge groups than in the control groups, whatever the breed. The increase in antibody titers between day 14 and day 28 was always higher in the challenge groups as compared to the control groups, this difference was highly significant in Ju-Chi and Nagoya (Table 2) and tended to be significant (*P* < 0.05) in all other breeds except Shek-Ki which never showed any difference in anti-IB titers between the challenged and the control groups.

**Table 2:** Effects of H6N1 challenge on antibody titers in different periods across breeds.

Breed	Period*	Control	Challenge
**Hsin-Yi**	IB14-IB0	6253 ± 499	5106 ± 499
	IB28-IB0	8119 ± 609	8769 ± 609
	IB28-IB14	1852 ± 726	3722 ± 738

**Ju-Chi**	IB14-IB0	4598 ± 553	3374 ± 541
	IB28-IB0	3474 ± 682^b^	5720 ± 608^a^
	IB28-IB14	-946 ± 564^b^	2408 ± 564^a^

**Hua-Tung**	IB14-IB0	4818 ± 877	3175 ± 784
	IB28-IB0	7149 ± 604	7461 ± 540
	IB28-IB14	2331 ± 766	4303 ± 699

**Quemoy**	IB14-IB0	3633 ± 508	3424 ± 469
	IB28-IB0	5261 ± 447	6362 ± 413
	IB28-IB14	1663 ± 502	2935 ± 463

**Shek-Ki**	IB14-IB0	2050 ± 687	1235 ± 615
	IB28-IB0	9464 ± 623	9809 ± 623
	IB28-IB14	7148 ± 938	8516 ± 884

**Nagoya**	IB14-IB0	6117 ± 548^a^	3165 ± 437^b^
	IB28-IB0	7038 ± 748	8718 ± 642
	IB28-IB14	580 ± 844^b^	5433 ± 695^a^

**SPF**	IB14-IB0	3205 ± 679	3063 ± 679
	IB28-IB0	3186 ± 962^b^	6118 ± 962^a^
	IB28-IB14	-19 ± 1107	3055 ± 1107

^*^IB14-IB0 represents the difference between IB14 and IB0, IB28-IB0 the difference between IB28 and IB0 and IB28-IB14 the difference between IB28 subtract IB14.

^a,b^ Means ± SE within a breed for a given stage with no common superscript differ significantly (*P* < 0.05).

Breed comparison showed that Quemoy had the highest antibody titers for IB0. Quemoy, Hsin-Yi and Nagoya had the highest values for IB14. These three breeds had still high values for IB28, but Shek-Ki had also high values for IB28, although it exhibited low values for IB0 and IB14. Thus, this breed was characterized by a late and strong response to IB vaccine, without any significant effect of the previous H6N1 challenge.

## Discussion and conclusions

### H6N1 LPAIV challenge effect

Mortality data and the increased antibody titers of challenge group at day 14 post-challenge showed that the challenge test had been effective. Estimation of breed effects may be affected by limited sample size, particularly for Shek-Ki, SPF and Nagoya. However, Quemoy had the largest sample size, and can be identified as the most resistant breed since it did not show any mortality after the challenge, and exhibited the most rapid immune response at day 7 post-challenge. At the opposite, Hsin-Yi can be identified as the most susceptible breed, with a rather high mortality (7 birds out of 26) in spite of the use of a low pathogenic virus.

### Breeds effect, vaccine efficacy and duration of immunity

Antibodies were detected for ND, IB and IBD at day 0 post-inoculation because all chickens had been vaccinated for ND, IB and IBD at earlier ages. Thus, the immune response following the inoculation at 11 weeks of age may be considered as a secondary immune response. Antibody titers at day 14 post-inoculation were significantly higher than at day 0, showing vaccine efficiency, except in the case of Nagoya and SPF for IBD vaccine where no change in antibody levels was observed.

Breed significantly affected immune response. Quemoy had high and rapid responses to the three vaccines and to H6N1 LPAVI challenge test, in contrast to Ju-Chi which had low immune response to vaccines and challenge test. Breed’s effect on the antibody titers at day 0 revealed differences in the duration of immunity to previous vaccines. Quemoy appeared to have a better immunity than other breeds, Shek-Ki had a slow response to ND and IB, and Ju-Chi presented lowest response to IBD. The better immune response of Quemoy is consistent with previous results [2].

### H6N1 LPAIV challenge effect on IB immune response

H6N1 LPAIV challenge had a negative effect on antibody levels to IB even before the vaccination at 11 weeks of age. Immune response to IB vaccine took place in H6N1 challenge groups with some delay (after 14 days). The negative relationship of H6N1 LPAIV challenge and IB vaccine response could be related to the fact that both viruses target the lung tissues. Recently, Haghighat-Jahromi et al. [4] showed that coinfection of H9N2 AI virus with IB live virus enhanced the virulence of H9N2 and increased the rate of mortality. In addition, Karimi-Madab et al. [5] showed that IB live vaccine could be an important risk factor resulting in enhanced virulence of H9N2 LPAIV in field conditions. Although these studies were focusing on broilers and H9N2 LPAIV, the present study shows also an interaction between IB and H6N1 LPAIV infection in some local chicken breeds. One could speculate that the type of local immune response induced by H6N1 LPAIV infection was not favourable for birds to make optimum IB-specific antibody response.

In conclusion, local breeds have different immune response to H6N1 LPAIV challenge and subsequent vaccines. The H6N1 LPAIV challenge influenced the response to subsequent vaccination against IB, but had no effect on ND and IBD subsequent vaccines.

## Competing interests

The authors declare that they have no competing interests.

## Authors' contributions

MTB and BB conceived the main idea of the study and helped to draft the manuscript. CSC carried out animal experiments, performed the statistical analysis and drafted the manuscript. YPL and CFC participated in the design of the study and provided experimental animals. PCC participated in the design of the study, and provided H6N1 virus. JWC participated in the design of the study, the analysis and the interpretation of the data. OC participated in the design of the study and helped to draft the manuscript. All authors contributed to the manuscript and all authors read and approved the final manuscript.
